# Validity and diagnostic performance of fluorescence optical imaging measuring synovitis in hand osteoarthritis: baseline results from the Nor-Hand cohort

**DOI:** 10.1186/s13075-020-02185-0

**Published:** 2020-05-01

**Authors:** Øystein Maugesten, Alexander Mathiessen, Hilde Berner Hammer, Sigrid Valen Hestetun, Tore Kristian Kvien, Till Uhlig, Sarah Ohrndorf, Ida Kristin Haugen

**Affiliations:** 1 Department of Rheumatology, Diakonhjemmet Hospital, Diakonveien 12, 0370 Oslo, Norway; 2 Faculty of Medicine, University of Oslo, Oslo, Norway; 3 Department of Rheumatology and Clinical Immunology, Charite Universitatsmedizin Berlin, Berlin, Germany

**Keywords:** Hand osteoarthritis, Xiralite, Inflammation, Synovitis, Ultrasound, MRI, Optical imaging, FOI

## Abstract

**Objective:**

Fluorescence optical imaging (FOI) demonstrates enhanced microcirculation in finger joints as a sign of inflammation. We wanted to assess the validity and diagnostic performance of FOI measuring synovitis in persons with hand OA, comparing it with magnetic resonance imaging (MRI)- and ultrasound-detected synovitis.

**Methods:**

Two hundred and twenty-one participants with hand OA underwent FOI and ultrasound (gray-scale synovitis and power Doppler activity) of the bilateral hands and contrast-enhanced MRI examination of the dominant hand. Fifteen joints in each hand were scored on semi-quantitative scales (grade 0–3) for all modalities. Four FOI images were evaluated: one composite image (Prima Vista Mode (PVM)) and three images representing phases of fluorescent dye distribution. Spearman’s correlation coefficients were calculated between sum scores of FOI, MRI, and ultrasound. Sensitivity, specificity, and area under the curve (AUC) were calculated for FOI using MRI or ultrasound as reference.

**Results:**

FOI did not demonstrate enhancement in the thumb base, and the joint was excluded from further analyses. FOI sum scores showed poor to fair correlations with MRI (rho 0.01–0.24) and GS synovitis sum scores (rho 0.12–0.25). None of the FOI images demonstrated both good sensitivity and specificity, and the AUC ranged from 0.50–0.61 and 0.51–0.63 with MRI and GS synovitis as reference, respectively. FOI demonstrated similar diagnostic performance with PD activity and GS synovitis as reference.

**Conclusion:**

FOI enhancement correlated poorly with synovitis assessed by more established imaging modalities, questioning the value of FOI for the evaluation of synovitis in hand OA.

## Background

Hand OA is a whole joint disease, affecting the cartilage, subchondral bone, synovium, and tendons [1]; however, the importance of inflammation in the hand OA pathogenesis remains debated. Ultrasound and magnetic resonance imaging (MRI) examinations have demonstrated a significant inflammatory burden in these patients, and synovitis is associated with pain [2] and radiographic progression on joint level [3, 4]. Inflammation has been of interest as a potential treatment target in recent OA trials. Whereas previous studies were not able to show clear clinically relevant effects [5], Kroon et al. recently showed significant effects of prednisolone on pain in persons with inflammatory hand OA, further supporting the role of inflammation in the pathogenesis of pain [6].

Valid and cost-efficient evaluation of inflammation will be important in future hand OA trials using synovitis as an inclusion criteria and/or outcome measure. Ultrasound and MRI are established modalities for assessing synovitis; however, they are limited by operator dependency and availability, contraindications, and higher cost, respectively. Fluorescence optical imaging (FOI) is a novel imaging modality using near-infrared light to demonstrate indocyanine green (ICG)-enhanced microcirculation in the region around finger joints as a sign of inflammation [7]. The method is without radiation, a scan of both hands takes only 6 min, and the device can be operated by trained health professionals.

Previous studies of patients with early and undifferentiated arthritis have shown moderate sensitivity (51–54%) and good specificity (81–87%) of composite FOI images [7, 8] using MRI-detected synovitis as reference, while another study found similar sensitivity and specificity in the proximal interphalangeal (PIP) joints in persons with rheumatoid arthritis (RA) [9]. The validity and diagnostic performance of FOI measuring synovitis have not been examined in persons with hand OA. Hence, we wanted to examine the frequency of FOI enhancement in persons with hand OA and assess whether FOI is correlated with MRI- and ultrasound-detected synovitis. Further, we wanted to investigate the diagnostic performance of FOI measuring synovitis in hand OA.

## Participants and methods

### Study participants

We included participants from the Nor-Hand study, an observational hand OA cohort from the rheumatology outpatient clinic at Diakonhjemmet Hospital, Oslo, Norway [10]. The participants were between 40 and 70 years old with proven hand OA by clinical and/or ultrasound examination and had no suspected diagnosis of systemic inflammatory joint diseases, psoriasis, or major somatic and/or psychiatric comorbidities. Further exclusion criteria are described elsewhere [10]. All participants signed informed consent, and the study was approved by the regional ethics committee.

### Fluorescence optical imaging (FOI)

**Fig. 1:**
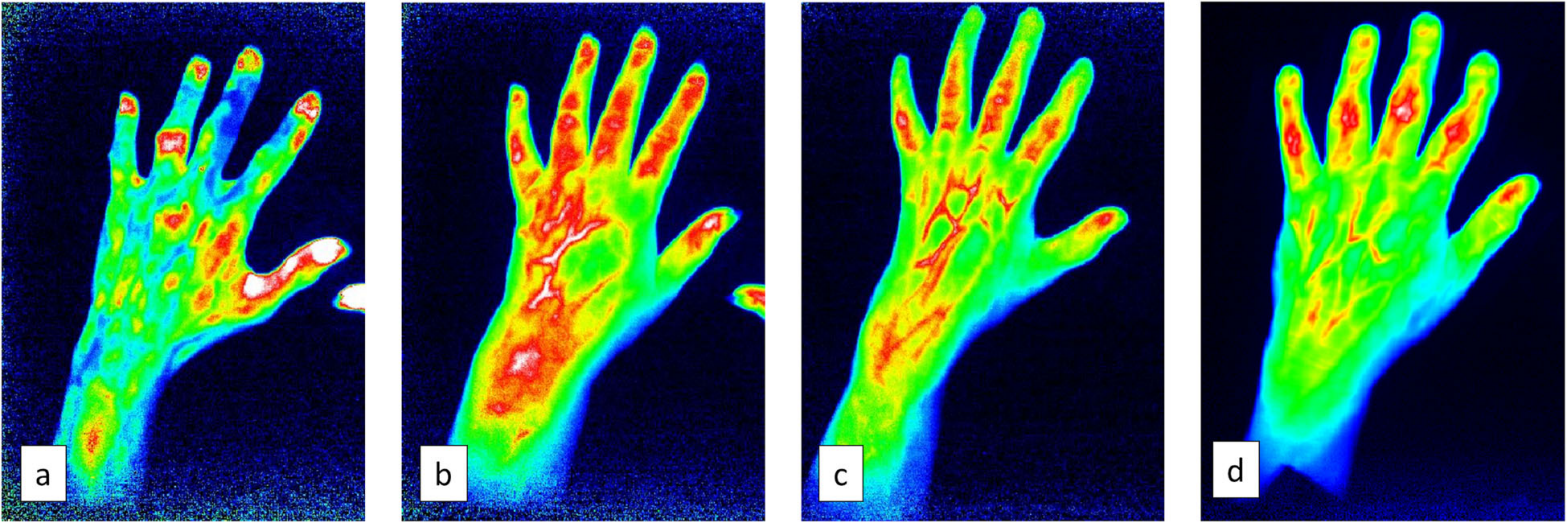
Examples of the different FOI activity score (FOIAS) images: phase 1 (**a**), phase 2 (**b**), phase 3 (**c**), and Prima Vista Mode (**d**)

The Xiralite®-system is the only FOI device available for clinical use in rheumatology. To perform the FOI scan, the patient receives an intravenous injection with a fluorescent dye (ICG pulsion, 0.1 mg/kg body weight) and have near-infrared light from light-emitting diodes (LED) projected down on the hands for 6 min. With a highly sensitive camera, 360 images (one/second) are produced, showing the flooding in, distribution, and washing out of the dye. All images can be scrolled through after the examination, and a composite picture (Prima Vista Mode (PVM)) from the 240 first images is automatically generated by the XiraView software. In short, four images are assessed with the FOI activity score (FOIAS): PVM and three images representing phases 1, 2, and 3 based on the distribution and washing out of the fluorescent dye in relation to the fingertips (Fig. 1). The distal interphalangeal (DIP) and proximal interphalangeal (PIP) including the 1st interphalangeal and metacarpophalangeal (MCP) joints and the thumb base were graded on 0–3 scales based on the color intensity and width of enhancement according to the FOIAS [8, 9, 11]. All FOI images were scored by one reader (SH) blinded for MRI and ultrasound results and all clinical data except age and sex. The reader was trained in assessing FOI images with good inter-reader reliability with an experienced reader (SO) and excellent intra-reader reliability for all phases except phase 1 (intraclass correlation coefficient for sum scores; PVM = 0.89, phase 1 = 0.10, phase 2 = 0.87, phase 3 = 0.89) in 21 patients [12]. Persons with known allergy to iodine or indocyanine, untreated hyperthyroidism with fT4 above 21 pmol/L and thyroid-stimulating hormone (TSH) below 0.5 mIE/L, poor liver function (transaminases above twice the upper reference limit), reduced kidney function (glomerular filtration rate below 40 mL/min), or pregnancy or breast-feeding did not perform the FOI scan.

### Magnetic resonance imaging (MRI)

Participants without contraindications underwent 1.5 T MRI (Siemens Aera, Germany) of the dominant hand. MRI was obtained mean (standard deviation (SD)) 9 (13.9) days after the FOI scan. The fingers and thumb base joints were covered by a 16-channel hand/wrist-coil and an intravenous contrast (Dotarem 279.3 mg/mL, 0.2 mL/kg body weight) was given. A T1-weighted volumetric interpolated breath-hold examination (VIBE) was reconstructed into three planes with 2.0 mm thickness, and the axial and sagittal planes were used for evaluation of synovitis [10]. The images were scored by a PhD student (ØM) trained in assessing synovitis in hand joints. Repeated training sessions with an experienced reader (IKH) were arranged prior to the calibration exercise with demonstration of atlases and evaluation of example images (*n* = 20). For calibration, 30 patients were scored separately in intervals of 13, 7, and 10 patients. Both readers scored the images until good inter-reader reliability (weighted kappa > 0.60) was obtained. For the last 10 patients, the scorers obtained a weighted kappa of 0.69. Joints with a difference of two or more grades and scores of 0 and 1 between the readers were reassessed and scored by consensus. For the remaining patients, the experienced reader (IKH) was consulted in case of uncertainties. The MRI reader was blinded for FOI and ultrasound results and all clinical data except age and sex. Synovitis in the DIP and PIP (incl. IP1) joints was assessed on a 0–3 scale according to the Hand OA MRI scoring system (HOAMRIS) [13], and the MCP joints were scored with same criteria as the PIP joints. All finger joints were assessed in the sagittal and axial planes and had to demonstrate consistent findings in 3 consecutive slices in both planes to qualify as MRI enhancement. The 1st carpometacarpal joint (CMC-1) and scaphotrapeziotrapezoidal (STT) joints were evaluated in the frontal and axial plane and evaluated using the TOMS atlas [14]. Flexor tenosynovitis was assessed according to the Oslo hand OA MRI scoring system (OHOA-MRI) and peritendinous inflammation along the extensor tendon was evaluated as absent/present [15].

### Ultrasound

A GE Logic S8 ultrasound machine with a linear 6–15 MHz probe preset for optimal gray-scale synovitis and power Doppler was used. The ultrasound examination was performed by a medical student trained by two experienced ultrasonographers (HBH, AM). A training session was arranged prior to study start with demonstration of the probe, normal B-mode musculoskeletal anatomy of the hand, and presentation of an atlas of synovitis grade 1–3 in the bilateral DIP and PIP including the first interphalangeal, MCP, and CMC-1 joints [16]. The hand joints were longitudinally scanned from the radial to the ulnar dorsal side, with additional transverse scanning in case of uncertainties. All joints were scored for gray-scale (GS) synovitis and power Doppler (PD) activity on semiquantitative 0–3 scales using the atlas from the training session as reference. The reader was blinded to MRI, FOI, and radiographic findings. The medical student and one of the experienced readers evaluated the 14 first patients together, and the medical student performed the remaining examinations independently. By the end of the data collection, a reliability exercise with the medical student and one ultrasonographer (AM) was performed, with consecutive enrollment of *n* = 10 patients with good inter-reader reliability (prevalence- and bias-adjusted kappa (PABAK) for GS in DIP/PIP (0.80) and CMC-1 joints (0.92) and power Doppler activity in DIP/PIP (0.85) and CMC-1 joints (0.92) [17].

### Conventional radiographs

Frontal images of bilateral hands were obtained with posterior-anterior view. One experienced reader (IKH) evaluated the DIP and PIP including the first interphalangeal, MCP, and CMC-1 according to the Kellgren Lawrence (KL) scale (grade 0–4) [18, 19] and Verbruggen Veys (VV) anatomical phase scoring system [20]. Erosive hand OA was defined as having at least one DIP or PIP joint(s) in the erosive or remodeling phase according to the VV anatomical phase scoring system. The reader demonstrated excellent intrareader reliability for both scoring systems with weighted kappa on 0.92 (KL) and kappa on 0.93 (erosive vs. non-erosive for the VV score).

### Statistics

Frequencies for different grades of FOI enhancement and synovitis detected by MRI and ultrasound were calculated and presented in histograms. Frequencies and trend of FOI enhancement in PVM across erosive vs. non-erosive and KL grades were assessed in cross tables and presented in histograms. We calculated Spearman’s correlations for sum scores of the dominant hand for MRI-detected synovitis and FOI and the bilateral hands for ultrasound-detected synovitis and FOI. For diagnostic performance, we calculated sensitivity, specificity, negative (NPV) and positive predictive values (PPV), and area under the curve (AUC) using either MRI or GS synovitis as reference. Percent agreement (PA) was calculated on FOI enhancement yes/no vs. GS/MRI synovitis yes/no. For all imaging modalities, joints missing due to amputation, trapeziectomy, or arthrodesis were imputed with an average value from the remaining joints in the same hand for sum scores, while they remained missing in calculations on frequencies and diagnostic performance. All results are presented for all joints together and for joint groups. Stratified analyses for persons with erosive hand OA vs. non-erosive hand OA were performed. Stata 14.0 was used for all the statistical analyses.

## Results

### Study population

Three hundred participants in the Nor-Hand cohort underwent ultrasound and radiographs of both hands. Among those, 246 participants performed MRI of the dominant hand with gadolinium contrast, and 253 participants performed FOI. One adverse event was reported due to subcutaneous administration of ICG, and the FOI images from this participant were excluded from further analyses. Finally, FOI images from two participants were excluded due to a lack of contrast enhancement. In total, 221 participants performed both FOI and MRI and were included for further analyses. The majority of participants were women, and a wide range in symptom severity, degree of inflammation, and structural damage was observed (Table 1).

**Table 1:** Baseline characteristics (*n* = 221)

Age, mean (SD) years	60.6 (6.2)
Women, *n* (%)	194 (88)
Body mass index, mean (SD) kg/m^2^	26.2 (4.7)
ACR criteria for hand OA, *n* (%)	203 (92)
Average NRS hand pain (range 0–10)*	3.7 (2.3)
HOAMRIS synovitis sum score DIP/PIP, mean (SD) [range 0–27]**	6.4 (4.8)
Patients with flexor tenosynovitis by MRI, *n* (%)	53 (24)
GS synovitis sum score DIP/PIP, mean (SD) [range 0–54]	4.4 (5.3)
PD activity sum score DIP/PIP, mean (SD) [range 0–54]	2.4 (4.3)
FOI PVM sum score, DIP/PIP, mean (SD) [range 0–54]	14.2 (7.3)
FOI phase 1 sum score, DIP/PIP, mean (SD) [range 0–54]	0.7 (2.5)
FOI phase 2 sum score, DIP/PIP, mean (SD) [range 0–54]	21.4 (9.7)
FOI phase 3 sum score, DIP/PIP, mean (SD) [range 0–54]	4.9 (5.7)
KL sum score, (DIP/PIP/MCP/CMC-1) mean (SD) [range 0–120]	28.8 (18.0)
Erosive hand OA, *n* (%)	74 (34)

*NRS pain on 220 patients, 1 missing

**Dominant hand

*ACR* American College of Rheumatology, *HOAMRIS* Hand OA MRI score, *KL* Kellgren-Lawrence, *DIP* distal interphalangeal, *PIP* proximal interphalangeal, *NRS* numeric rating scale, *OA* osteoarthritis

### Frequency distribution of synovitis according to FOI, MRI, and ultrasound

For GS synovitis and PD activity, 27 joints were missing due to amputation, trapeziectomy, arthrodesis, or unknown reasons. Five joints were missing due to trapeziectomy, arthrodesis, or amputation on MRI of the dominant hand. One phase 1 image, seven phase 2 images, and eight phase 3 images were excluded from analyses due to difficulties defining phases, i.e., no clear descending of the white from fingertips (phase 1) and white (phase 2) or red (phase 3) pixels persisting in fingertips.

**Fig. 2:**
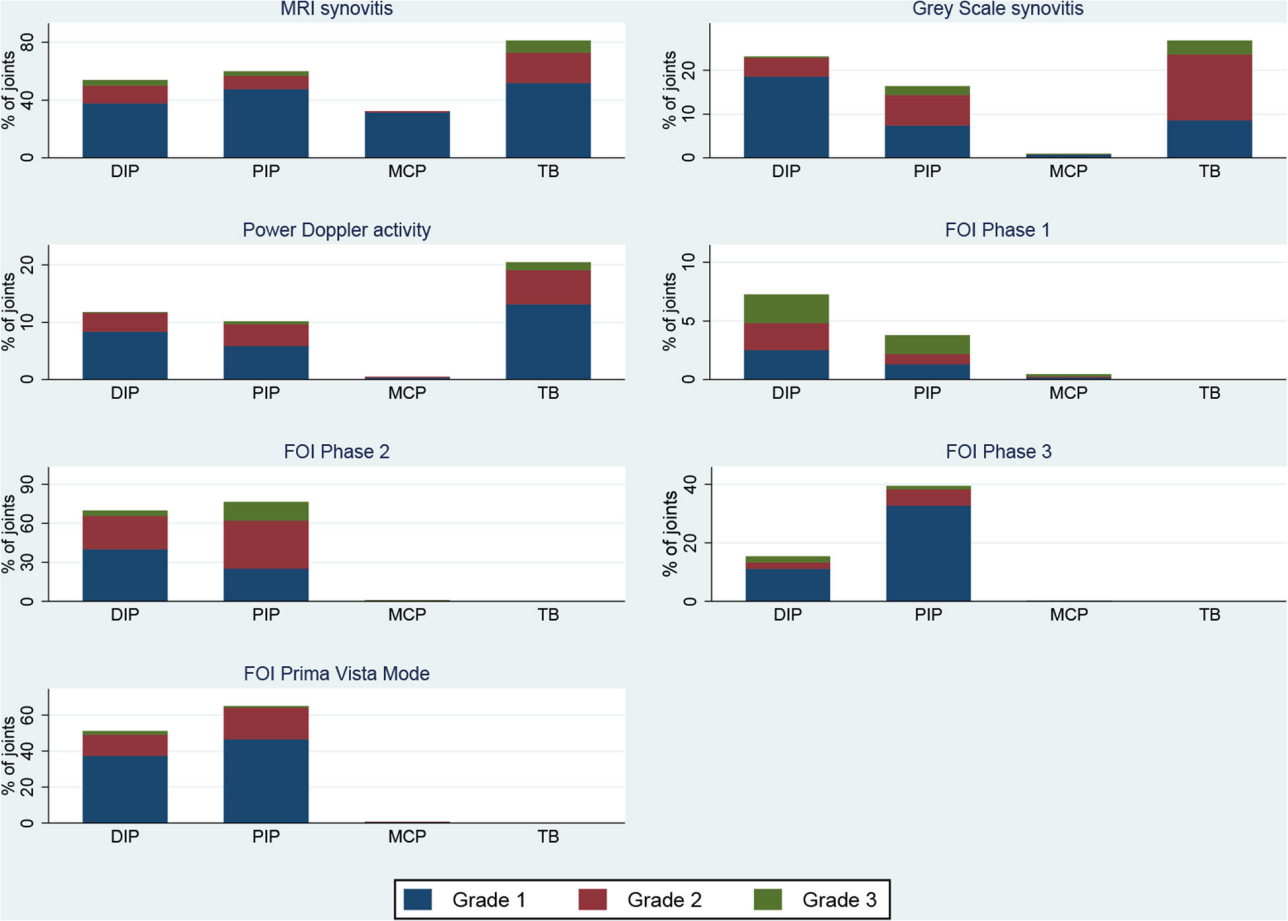
Frequency distribution of FOI enhancement, MRI and gray-scale synovitis and power Doppler activity in hand OA patients. MRI, magnetic resonance imaging; DIP, distal interphalangeal joints; PIP, proximal interphalangeal joints; MCP, metacarpophalangeal joints; TB, thumb base. ^1^MRI findings from dominant hand only, FOI and ultrasound from bilateral hands.^2^The thumb base (TB) includes CMC-1 and/or STT synovitis for MRI and CMC-1 synovitis for ultrasound. The TB region is assessed as a whole for FOI, as the CMC-1 and STT joint cannot be distinguished

None of the participants demonstrated FOI enhancement of the thumb base, while 81% of the participants had MRI-defined synovitis in this area (CMC-1 and/or STT). The CMC-1 joint was more frequently affected (69%) than the STT joint (54%). Ultrasound of the CMC-1 joint demonstrated less synovitis than MRI (gray-scale synovitis 26%, power Doppler activity 19%) (Fig. 2). Due to the lack of FOI enhancement in the thumb base, it was not included in further analyses. Only three MCP1 joints showed any FOI enhancement, and MRI was the only modality showing frequent findings in the MCP joints (32% of joints, predominantly grade 1). While MRI and FOI (PVM and phases 2 and 3) detected more synovitis and enhancement in the PIP joints than in the DIP joints, GS synovitis and PD activity and FOI phase 1 demonstrated more activity in the DIP joints.

None of the participants demonstrated MRI-enhanced peritendinous inflammation along the extensor tendon. Fifty-three participants had flexor tenosynovitis in one or more fingers, and the majority (*n* = 46) demonstrated grade 1 tenosynovitis adjacent to the MCP joint. Flexor tenosynovitis was not included in further analysis due to its localization on the palmar aspect of the hand and thus not detectable via FOI. When assessing frequency of FOI enhancement in PVM according to VV and KL scores, we found a significant trend for higher proportion of joints with FOI enhancement in joints with severe KL and VV grades (Online supplementary figure 1).

### Correlations between FOI, ultrasound, and MRI

**Fig. 3:**
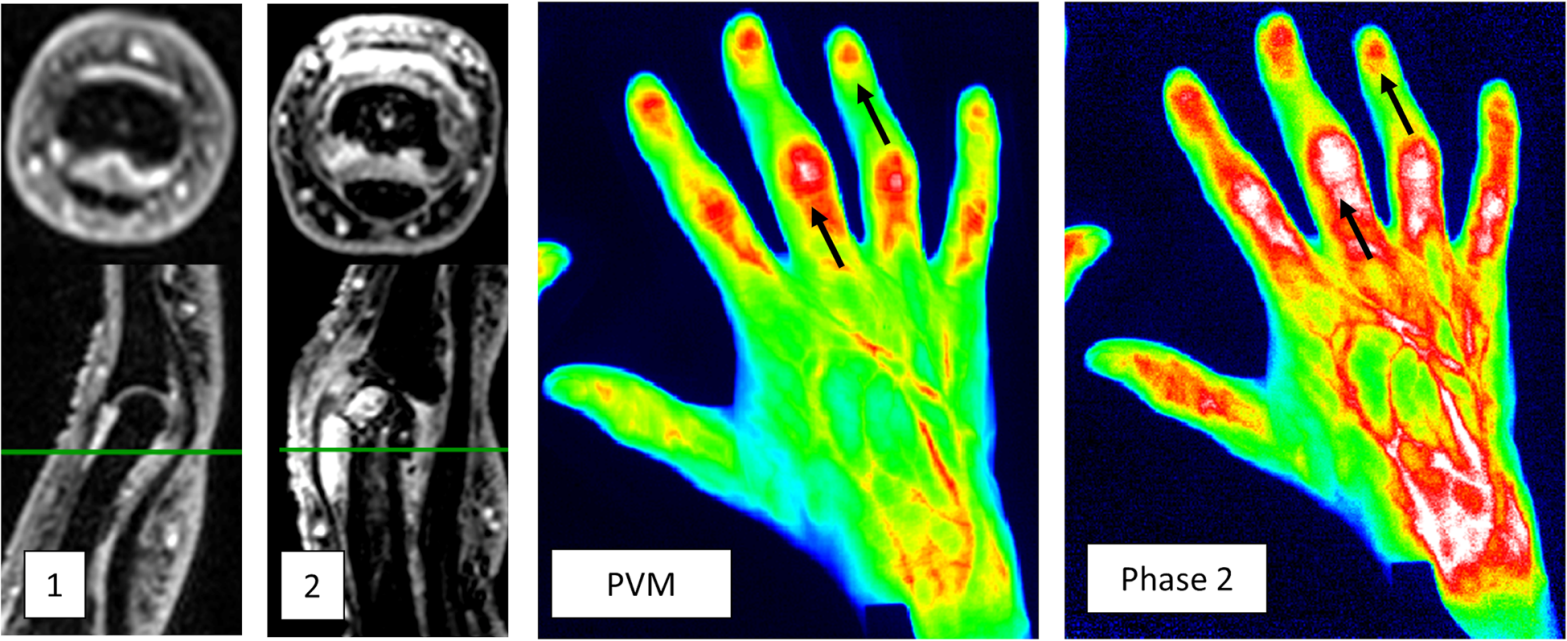
MRI synovitis grade 2 on axial and sagittal plane in DIP 4 (1) and grade 3 in PIP 3 (2) compared with FOI PVM and FOI phase 2 with good agreement in PIP joint and poor agreement in DIP joint. MRI, magnetic resonance imaging; FOI, fluorescence optical imaging; PVM, Prima Vista Mode; DIP, distal interphalangeal joint; PIP, proximal interphalangeal joint

Good correlations were found between MRI and GS synovitis for all joint groups except in the MCP joints (Table 2). Similarly, GS synovitis and PD activity demonstrated good to very good correlations for all joint groups. Overall, the correlations between FOI and MRI were poor to fair, while FOI was poorly correlated with GS synovitis. The strongest correlation with MRI was found for PVM in the PIP joints with Spearman’s rho of 0.32, while the DIP joints had consistently the weakest correlations ranging from 0.00 to 0.14 (Table 2, Fig. 3).

**Table 2:** Spearman’s correlations for synovitis sum scores between MRI, ultrasound, and FOI

Variable 1	Variable 2	All joints	DIP	PIP	MCP
MRI*	PVM*	0.23	0.09	0.32	0.17
MRI*	Phase 1*	0.01	0.00	0.01	− 0.04
MRI*	Phase 2*	0.24	0.14	0.31	− 0.01
MRI*	Phase 3*	0.19	0.09	0.24	0.07
GS	PVM	0.15	0.07	0.26	0.20
GS	Phase 1	0.12	0.15	0.04	0.13
GS	Phase 2	0.25	0.15	0.30	0.13
GS	Phase 3	0.22	0.06	0.27	0.17
MRI*	GS*	0.58	0.45	0.60	− 0.04
MRI*	PD*	0.45	0.35	0.47	− 0.02
GS	PD	0.79	0.70	0.85	0.79

*Dominant hand

*MRI* magnetic resonance imaging, *GS* gray-scale ultrasound, *PD* power Doppler, *FOI* fluorescence optical imaging, *PVM* FOI Prima Vista Mode, *Phase 1* FOI phase 1, *Phase 2* FOI phase 2, *Phase 3* FOI phase 3, *DIP* distal interphalangeal joint, *PIP* proximal interphalangeal joint, *MCP* metacarpophalangeal joint

### Diagnostic performance of FOI measuring synovitis

Using MRI and GS synovitis as reference, FOI phase 1 demonstrated the highest specificity, with corresponding very low sensitivity (Table 3). FOI PVM and phase 2 had consistently the highest sensitivities with both MRI and GS synovitis as reference, with values ranging from 48% to 69%. FOI reached high NPV with GS synovitis as reference, suggesting that joints with no FOI enhancement were unlikely to have GS synovitis. However, presence of FOI enhancement did not consistently correspond with presence of GS synovitis, demonstrated by low PPV values. GS synovitis was less prevalent than MRI synovitis, which affected the results considerably. Using MRI instead of ultrasound as reference, FOI demonstrated higher PPV and lower NPV. However, improvement of sensitivity, specificity, and AUC was found for FOI when presence of MRI synovitis was increased to grade 2 or more (Online supplementary table 1, online supplementary figure 2). The agreement between FOI (enhancement yes/no) and MRI (synovitis yes/no) ranged from 53 to 61% while the same values for ultrasound (synovitis yes/no) ranged from 57 to 89%. Using PD activity as reference, the diagnostic performance of FOI was similar to the results when GS synovitis was used as reference (data not shown).

**Table 3:** Diagnostic performance of FOI measuring synovitis using MRI and GS synovitis as reference

FOI	FOI+/MRI+	FOI−/MRI-	Sens.	Spec.	PPV	NPV	AUC	PA
PVM	698/1456	1180/1635	0.48	0.72	0.61	0.61	0.61	61
Phase 1	22/1442	1621/1635	0.02	0.99	0.61	0.53	0.50	53
Phase 2	814/1408	984/1585	0.58	0.62	0.58	0.62	0.60	60
Phase 3	332/1407	1408/1572	0.24	0.90	0.67	0.57	0.57	58
FOI	FOI+/GS+	FOI−/GS−	Sens.	Spec.	PPV	NPV	AUC	PA
PVM	407/688	3510/5473	0.59	0.64	0.17	0.93	0.62	64
Phase 1	16/680	5412/5454	0.02	0.99	0.28	0.89	0.51	88
Phase 2	461/667	2977/5299	0.69	0.56	0.17	0.94	0.63	58
Phase 3	152/664	4545/5273	0.23	0.86	0.17	0.90	0.56	79

*FOI* fluorescence optical imaging, *PVM* FOI Prima Vista Mode, *Phase 1* FOI phase 1, *Phase 2* FOI phase 2, *Phase 3* FOI phase 3, *MRI* magnetic resonance imaging, *GS* gray-scale ultrasound, *Sens.* sensitivity, *Spec.* specificity, *PPV* positive predictive value, *NPV* negative predictive value, *PA* percent agreement, *AUC* area under the curve

### Results from subgroup analysis

Correlation analyses were repeated for participants with erosive hand OA without consistent improvements in the correlations between FOI, MRI, and GS synovitis. Further, the diagnostic performance of FOI measuring synovitis with MRI and ultrasound as reference was similar in erosive hand OA and non-erosive hand OA patients (data not shown).

## Discussion

This is the first study to investigate the validity and diagnostic performance of FOI in persons with hand OA. To our knowledge, the Nor-Hand study is also the largest clinical study to date comparing FOI with MRI and ultrasound.

Our hand OA patients demonstrated a significant inflammatory burden with a high percentage of joints with MRI- and ultrasound-detected synovitis, with the DIP, PIP, and thumb base joints most frequently affected. FOI demonstrated most enhancement in DIP and PIP joints, whereas no enhancement was detected in the thumb base despite inflammation in these joints being highly prevalent on both MRI and ultrasound. FOI enhancement in the thumb base has not been detected in previous studies on FOI, and we hypothesize that the CMC-1 and STT joints are located too deep to be visualized by the limited tissue penetration of the FOI device. This represents an important limitation for the use of FOI in hand OA. The development of a 3D FOI device with pairing of lateral, medial, palmar, and dorsal images would possibly give a more complete representation of the inflamed joint and could therefore improve the correlation to MRI and ultrasound in persons with hand OA.

FOI showed poor to fair correlation with MRI and ultrasound in our cohort. In contrast, Fischer et al. found strong correlation between MRI and FOI in five RA patients with a similar near-infrared optical imaging device [21], and Werner et al. demonstrated moderate correlation between gray-scale synovitis and FOI (rho = 0.40) in patients with arthritis using the Xiralite® scanner [7]. Regarding diagnostic performance, we found moderate to very good specificities and poor to moderate sensitivities for FOI using MRI-detected synovitis as reference, with the best specificity in FOI phase 2 (99%) with corresponding low sensitivity (2%), suggesting substantial noise and false positive findings. Previous studies on RA and undifferentiated arthritis have demonstrated better specificity and sensitivity for FOI, particularly for phase 1 [7–9, 11, 22]. Phase 1 has been suggested to demonstrate active inflammation [23] and might explain the higher sensitivity of this phase in persons with RA rather than hand OA. This is supported by the finding of fewer joints with PD activity in our cohort, with mean sum score of 2.4 in DIP and PIP joints in the bilateral hands. In comparison, a group of 431 RA patients demonstrated a mean sum score of PD activity of 4.8 in the wrist, MCP 1–5 and PIP 2–3 of the dominant hand [24].

The percent agreement was better between FOI (enhancement yes/no) and ultrasound (synovitis yes/no) than FOI and MRI, most likely due to the high prevalence of low-grade MRI synovitis in our cohort. It is debated whether MRI grade 1 synovitis actually represents pathology or rather is a normal finding [25], and we found improved values when assessing the diagnostic performance and percent agreement of FOI with MRI-defined synovitis grade 2 and higher as reference.

Despite our findings of poor correlations and diagnostic performance, FOI enhancement has previously corresponded to histological synovitis in animal models with induced arthritis [26]. Interestingly, we found more FOI enhancement in joints with increasing KL and VV grade, especially in the erosive joints. Bone remodeling with increased vascularity of the bone in OA joints may have affected the enhancement, although it is unknown whether these signals can be detected by FOI. Further, it is unlikely that tenosynovitis has affected the results as the low degree of flexor tenosynovitis detected on MCP level in our cohort is located too deep to be detected by FOI, comparable to the aforementioned thumb base. Additionally, no participants had peritendinous inflammation along the extensor tendon. FOI enhancement in our participants might represent an extraarticular hypervascularity due to inflamed subcutaneous tissue; however, we did not specifically look for this feature when assessing the MRI images.

Poor agreement between FOI and MRI might also be a question of scoring method. The FOI reader in our study demonstrated good reliability with an experienced reader for phase 2 and 3 and PVM; however, phase 1 showed remarkably low inter-reader reliability (ICC = 0.10). Readers define phases 1, 2, and 3 from preset criteria and might assess different images. In a recently published paper, we found low reliability for phase 1 in both hand OA and RA patients and we hypothesized that the low agreement in phase 1 was due to rapid changes in the beginning of the FOI image sequence, while phase 2 and phase 3 had good reliability despite readers assessing images within a broad range [27]. Ultimately, the FOIAS might not be the best scoring method for analyzing the 360 images in persons with hand OA. FOI and its varying degrees of enhancement seems particularly suited for developing an automated algorithm for scoring affected joints through, e.g., machine learning, and might improve the diagnostic performance and validity of FOI in persons with hand OA. This study has several limitations. First, our participants were recruited from a rheumatology outpatient clinic, making it difficult to generalize the results to persons with hand OA in primary health care. Secondly, FOI was performed approximately 2 weeks prior to MRI. As low-grade MRI-defined synovitis might fluctuate and represent a normal finding, images should have been acquired on the same day in order to make FOI and MRI fully comparable. However, the ultrasound exam was conducted on the same day as the FOI exam and demonstrated good correlation (*r* = 0.58) with the MRI findings.

## Conclusion

To conclude, we found poor to fair correlation between FOI enhancement and MRI- and ultrasound-detected synovitis in persons with hand OA. None of the FOI phases or PVM demonstrated both good sensitivity and specificity. Although a frequent manifestation of hand OA, FOI was not able to detect synovitis in the thumb base. Our cohort demonstrated low-grade inflammation with less vascularization, which might explain the poor results compared with previous FOI studies on systemic inflammatory joint diseases. With the current scoring method and technology available, we conclude that MRI and ultrasound perform better than FOI for the assessment of inflammation in hand OA.

## Supplementary information

**Additional file 1 : Figure S1.** Distribution of FOI PVM enhancement in joints with increasing degree of osteoarthritis.

**Additional file 2 : Table S1.** The diagnostic performance of FOI measuring synovitis using MRI grade 2 & 3 as reference.

**Additional file 3 : Figure S2.** ROC curves showing the AUC of FOI PVM using a) MRI grade 1-3 and b) MRI grade 2-3 as reference.

## Data Availability

The datasets during and/or analyzed during the current study available from the corresponding author on reasonable request.

## Abbreviations

ACR: American College of Rheumatology
AUSCAN: Australian and Canadian Hand Index
CI: Confidence interval
CMC-1: 1st carpometacarpal joint
CR: Conventional radiography
DIP: Distal interphalangeal joints
FOI: Fluorescence optical imaging
HOAMRIS: Hand OA MRI score
ICC: Intraclass correlation coefficient
IP: Interphalangeal
IQR: Interquartile range
JSN: Joint space narrowing
KL: Kellgren Lawrence
MCP: Metacarpophalangeal joints
MRI: Magnetic resonance imaging
OA: Osteoarthritis
PA: Posterior-anterior
PIP: Proximal interphalangeal joint
RA: Rheumatoid arthritis
SD: Standard deviation
STT: Scaphotrapeziotrapezoid
