# Correction to: Validity and diagnostic performance of fluorescence optical imaging measuring synovitis in hand osteoarthritis: baseline results from the Nor-Hand cohort

**DOI:** 10.1186/s13075-021-02469-z

**Published:** 2021-03-17

**Authors:** Øystein Maugesten, Alexander Mathiessen, Hilde Berner Hammer, Sigrid Valen Hestetun, Tore Kristian Kvien, Till Uhlig, Sarah Ohrndorf, Ida Kristin Haugen

**Affiliations:** 1 Department of Rheumatology, Diakonhjemmet Hospital, Diakonveien 12, 0370 Oslo, Norway; 2 Faculty of Medicine, University of Oslo, Oslo, Norway; 3 Department of Rheumatology and Clinical Immunology, Charite Universitatsmedizin Berlin, Berlin, Germany

**Correction to: Arthritis Res Ther 22, 98 (2020)**

**https://doi.org/10.1186/s13075-020-02185-0**

Following publication of the original article [1], the authors reported an error in Fig. 2 and the other is in the supplementary Figure 1.

1. In Fig. 2 the percentage of joints with MRI-defined synovitis grade 1–3 in the thumb base is reported as 81%. The correct number is 61%. As reported under “Results, Frequency distribution of synovitis according to FOI, MRI, and ultrasound” 81% of the participants demonstrated MRI enhancement in the CMC-1 and/or STT joint. However, 61% of the total STT and CMC-1 joints examined demonstrated MRI-defined synovitis.

**Fig. 2:**
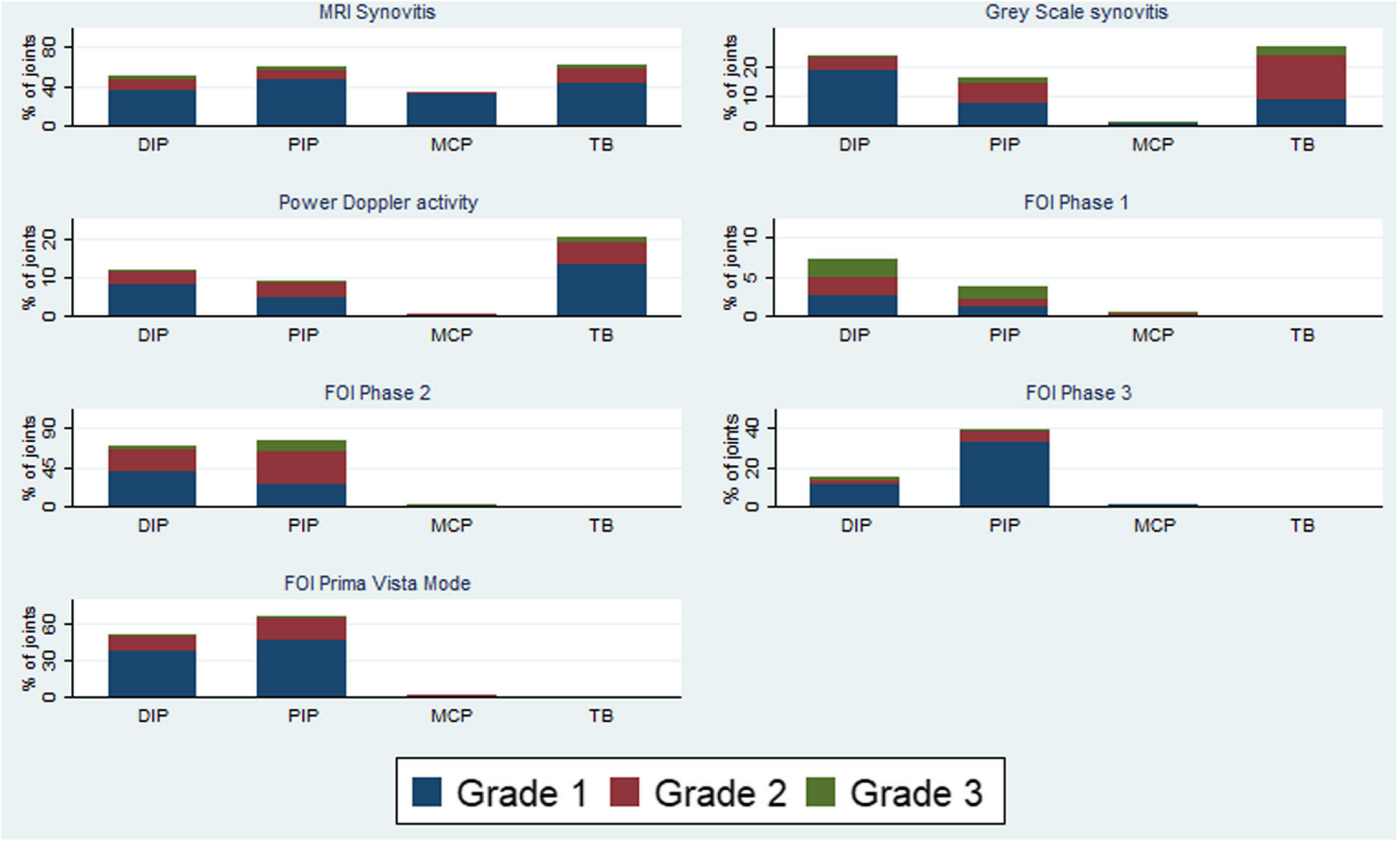
Frequency distribution of FOI enhancement, MRI and gray-scale synovitis and power Doppler activity in hand OA patients. MRI, magnetic resonance imaging; DIP, distal interphalangeal joints; PIP, proximal interphalangeal joints; MCP, metacarpophalangeal joints; TB, thumb base. ^1^MRI findings from dominant hand only, FOI and ultrasound from bilateral hands. ^2^The thumb base (TB) includes CMC-1 and/or STT synovitis for MRI and CMC-1 synovitis for ultrasound. The TB region is assessed as a whole for FOI, as the CMC-1 and STT joint cannot be distinguished

2. Supplementary Figure 1: Erosive and remodeling phase is reported in two identical columns. The correct figure should have only one of the two columns representing joints in erosive and remodeling phase.”

The original article has been corrected.

## Supplementary Information

**Additional file 1:** **Figure S1.** Distribution of FOI PVM enhancement in joints with increasing degree of osteoarthritis.
