# Supplementary figures and images for: Validity and diagnostic performance of fluorescence optical imaging measuring synovitis in hand osteoarthritis: baseline results from the Nor-Hand cohort
Source: Arthritis Res Ther. 2020 May 1;22:98. doi: 10.1186/s13075-020-02185-0 (PMC7193370; doi:10.1186/s13075-020-02185-0)

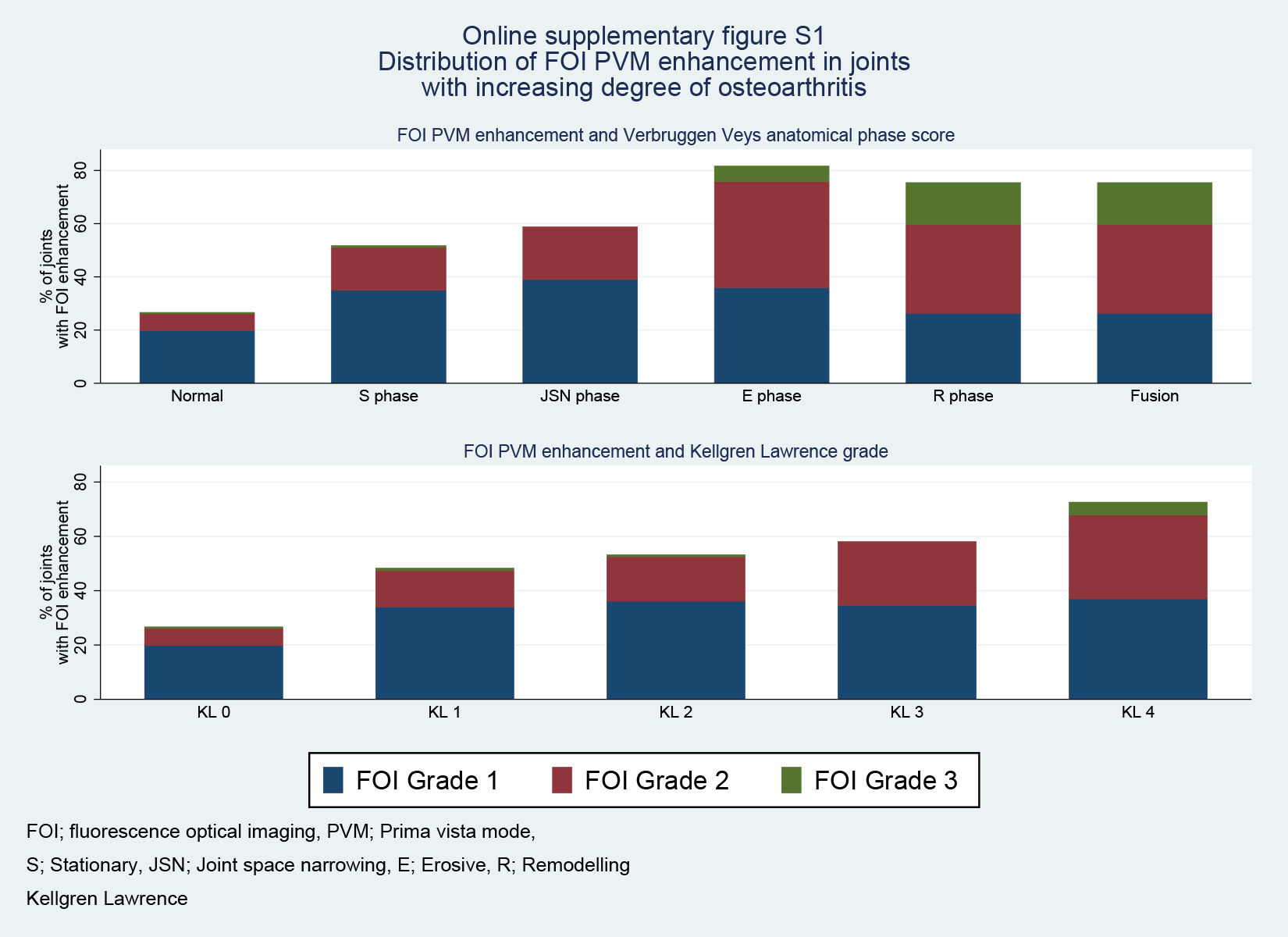

Supplement: Supplementary file 1 — Additional file 1 : Figure S1. Distribution of FOI PVM enhancement in joints with increasing degree of osteoarthritis. [file 13075_2020_2185_MOESM1_ESM.tif]

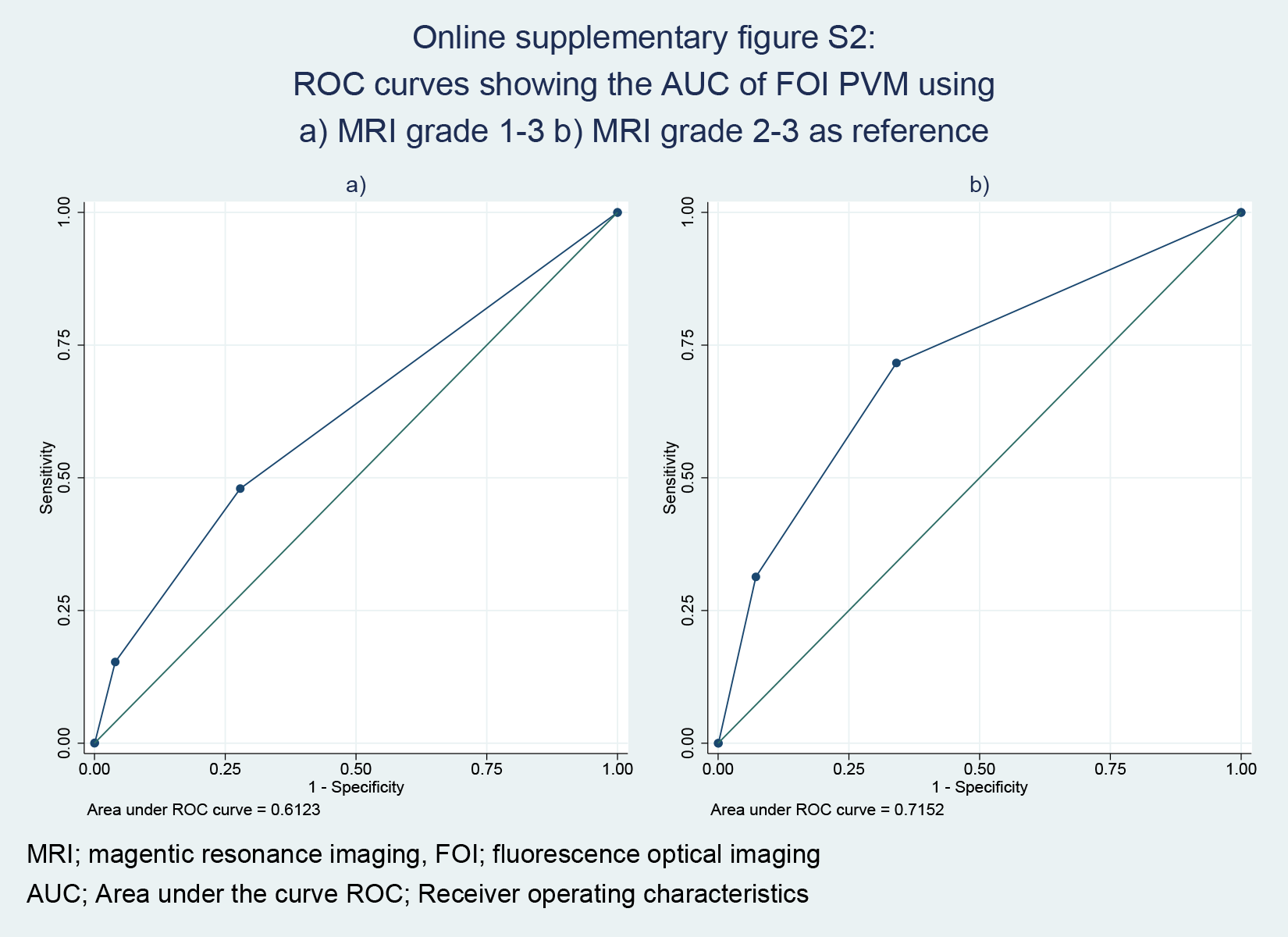

Supplement: Supplementary file 3 — Additional file 3 : Figure S2. ROC curves showing the AUC of FOI PVM using a) MRI grade 1-3 and b) MRI grade 2-3 as reference. [file 13075_2020_2185_MOESM3_ESM.tif]
