# Supplementary material for: Validity and diagnostic performance of fluorescence optical imaging measuring synovitis in hand osteoarthritis: baseline results from the Nor-Hand cohort
Source: Arthritis Res Ther. 2020 May 1;22:98. doi: 10.1186/s13075-020-02185-0 (PMC7193370; doi:10.1186/s13075-020-02185-0)
Supplement: Supplementary file 2 — Additional file 2 : Table S1. The diagnostic performance of FOI measuring synovitis using MRI grade 2 & 3 as reference. [file 13075_2020_2185_MOESM2_ESM.docx]

Online supplement Table S1: The diagnostic performance of FOI measuring synovitis using MRI grade 2 & 3 as reference.

| FOI | FOI+/MRI+ | FOI-/MRI- | Sens. | Spec. | PPV | NPV | AUC | PA |
| --- | --- | --- | --- | --- | --- | --- | --- | --- |
| PVM | 192/268 | 1862/2823 | 0.72 | 0.66 | 0.17 | 0.96 | 0.72 | 66 |
| Phase 1 | 10/265 | 2786/2812 | 0.04 | 0.99 | 0.28 | 0.92 | 0.51 | 91 |
| Phase 2 | 213/260 | 1531/2733 | 0.82 | 0.56 | 0.15 | 0.97 | 0.74 | 58 |
| Phase 3 | 111/260 | 2334/2719 | 0.43 | 0.86 | 0.22 | 0.94 | 0.64 | 82 |

*FOI=Fluorescence optical imaging, PVM=FOI Prima Vista Mode, Phase 1= FOI Phase 1, Phase 2= FOI Phase 2, Phase 3= FOI Phase 3, MRI= Magnetic Resonance Imaging, GS= Grey Scale Ultrasound, Sens.= sensitivity, Spec.= Specificity, PPV= Positive Predictive Value, NPV= Negative Predictive Value, PA= Percent Agreement, AUC= Area under the curve.*
