# Supplementary figures and images for: Correction to: Validity and diagnostic performance of fluorescence optical imaging measuring synovitis in hand osteoarthritis: baseline results from the Nor-Hand cohort
Source: Arthritis Res Ther. 2021 Mar 17;23:87. doi: 10.1186/s13075-021-02469-z (PMC7968308; doi:10.1186/s13075-021-02469-z)

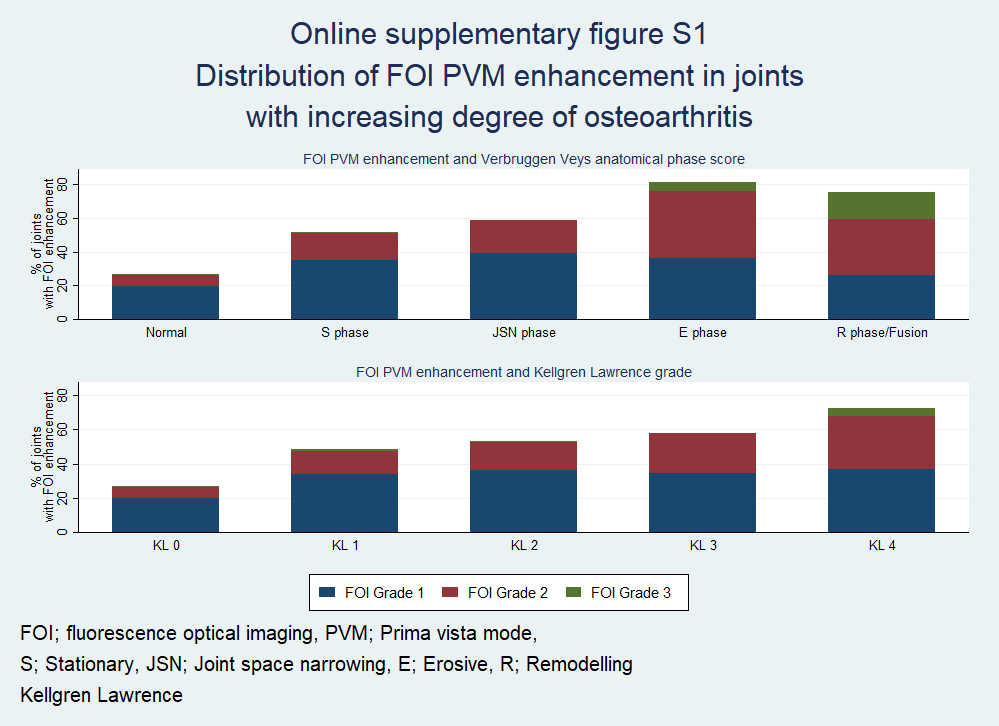

Supplement: Supplementary file 1 — Additional file 1: Figure S1. Distribution of FOI PVM enhancement in joints with increasing degree of osteoarthritis. [file 13075_2021_2469_MOESM1_ESM.tif]
